# Yiqi Huayu Jiedu Decoction Inhibits the Invasion and Metastasis of Gastric Cancer Cells through TGF-*β*/Smad Pathway

**DOI:** 10.1155/2017/1871298

**Published:** 2017-04-30

**Authors:** Ting-Ting Wu, Jun Lu, Pei-Qiu Zheng, Shen-Lin Liu, Jian Wu, Wei Sun, Qing-Min Sun, Nai-Xia Ma, Xue-Lian Ding, Min Chen, Xi Zou

**Affiliations:** ^1^The Affiliated Hospital of Nanjing University of TCM, Jiangsu Province Hospital of TCM, Nanjing 210029, China; ^2^No. 1 Clinical Medical College, Nanjing University of Chinese Medicine, Nanjing, Jiangsu 210023, China

## Abstract

*Background.* Yiqi Huayu Jiedu Decoction (YHJD) can obviously improve the quality of life of those patients with gastric cancer and prolong their survival.* Methods*. In vitro experiments, we observe YHJD's effect on the cells' proliferation by MTT assay. Cell adhesion assay, wound-healing assay, and Transwell invasion assay serve to detect its influence on cells' adhesion, migration, and invasion, respectively. Inhibitor (10 *μ*M/L of SB431542) and activator (10 ng/mL of TGF-*β*) of TGF-*β*/Smad pathway were used to estimate whether YHJD's impact on the biological behavior of gastric cancer cells was related to TGF-*β*/Smad pathway. In in vivo studies, YHJD was administered to the nude mice transplanted with gastric cancer to observe its effect on the tumor. Western blotting and immunohistochemical assay were used to test relevant cytokines of TGF-*β*/Smad pathway and epithelial-mesenchymal transition (EMT) in MGC-803 cells and the tumor bearing nude mice.* Results.* YHJD inhibited proliferation, adhesion, migration, and invasion of MGC-803 gastric cancer cells in vitro. In in vivo studies, YHJD reduced the volume of the transplanted tumors. It also enhanced the expression of E-cadherin and decreased the levels of N-cadherin, TGF-*β*, Snail, and Slug in both MGC-803 cells and the transplanted tumor by western blot assay. The immunohistochemical assay revealed that YHJD raised E-cadherin in the tumors of the mice; on the contrary, the expression of N-cadherin, Twist, vimentin, TGF-*β*R I, p-Smad2, p-Smad3, Snail, and Slug reduced.* Conclusion*. YHJD can effectively inhibit the invasion and metastasis of gastric cancer cells. The mechanism may be related to TGF-*β*/Smad pathway.

## 1. Introduction

Gastric cancer is one of the most common malignant tumors in China, which is identified as the leading cause of cancer death [1]. A survey demonstrates that the primary reason for the death of patients with gastric cancer is recurrence and distant metastasis of the tumor. The relapse rates of patients with gastric cancer who have undergone surgery are 50% within one year and 70% within two years, while 80% of the patients were found to exhibit local or distant metastasis in autopsy after death [2, 3]. Therefore, drugs against the growth and metastasis of gastric cancer are urgently required.

Natural medicine is an important source of antitumor chemotherapy, and to find effective ingredients from natural medicine is one of the important strategies of antitumor drug development today. Yiqi Huayu Jiedu Decoction (YHJD) is comprised of Shenghuangqi (Radix Astragali), Dangshen (Radix et Rhizoma Salviae Miltiorrhizae), Sanleng (Rhizoma Sparganii), Ezhu (Rhizoma Curcumae), Danshen (Radix et Rhizoma Salviae Miltiorrhizae), Yinchen (Herba Artemisiae Scopariae), Banzhilian (Herba Scutellariae Barbatae), Dijincao (Herba Euphorbiae Humifusae), and Baqia (Rhizoma Smilacis Chinae), which is a summary of 40 years of clinical experience of the nationally renowned TCM (traditional Chinese medicine) doctor Professor Liu Shenlin. Clinical study on large samples has shown that YHJD can obviously improve the quality of life of patients and prolong their survival, but it is rare to see researches into its underlying molecular mechanisms. However, formulae have their specific and complicated effects through a variety of pathways and targets due to multiple compositions and complicated metabolic processes. Thus, the underlying molecular mechanisms associated with the therapeutic potential for gastric cancer remain unknown.

The first step of tumor metastasis is the weakening of the intercellular adhesive ability, the increase of the intercellular invasive ability, and finally falling off of tumor cells from the primary lesion. Epithelial-mesenchymal transition (EMT) plays a crucial role during cancer invasion and metastasis [4, 5]. The TGF-*β*/Smad pathway which is activated by transforming growth factor-*β* (TGF-*β*) is the main mechanism of EMT [6, 7]. In addition, studies have found that an increased expression of TGF-*β* is found in various human cancers, including gastric cancer [8, 9]. Thus, targeting TGF-*β* can be considered as a potential strategy in the prevention and treatment of gastric cancer.

This study aims to reveal that YHJD can inhibit the invasion and metastasis of gastric cancer through TGF-*β*/Smad pathway in vivo and in vitro.

## 2. Materials and Methods

### 2.1. Cells and Animals

Human gastric cancer cell line MGC-803 was under routine culture at the Centre Laboratory of Jiangsu Provincial Hospital of TCM. It was cultured in RPMI-1640 medium (HyClone, Thermo Scientific, USA) supplemented with 10% fetal calf serum (Evergreen Company, Hangzhou, China). The cells were incubated at 37°C, 5% CO_2_, and saturated humidity atmosphere.

24 BALB/c mice (number 201605969) were purchased from Charles River, Beijing, China. The mice were half male and half female, 4 weeks old, weighing 18–22 g. They were fed in SPF environment with regular indoor ultraviolet radiation. Their cage, litter, feeds, and water were strictly sterilized.

### 2.2. Yiqi Huayu Jiedu Decoction (YHJD)

All the medical herbs were sourced from Jiangsu Province Hospital of TCM. Herbs (165 g), including 30 g Shenghuangqi, 30 g Dangshen, 15 g Sanleng, 15 g Ezhu, 15 g Danshen, 15 g Yinchen, 15 g Banzhilian, 15 g Dijincao, and 15 g Baqia, were boiled with 1650 mL of distilled water, refluxed, and extracted. 4 g/mL herb water extract was obtained from the mixed decoctions and then filtered through a 0.2 *μ*m filter. The extract was stored at −20°C until use.

### 2.3. MTT Assay

Cells in the logarithm phase were seeded in 96-well plates at the density of 6 × 10^3^/well.

When the cells were adherent to the walls, the experimental groups were divided into control group, TGF-*β* group, TGF-*β* + YHJD group, SB431542 group, SB431542 + YHJD group, and YHJD group, respectively. The control group were add equal amount of complete medium: TGF-*β* at 10 ng/mL (Peprotech Bio Co., Ltd., China), SB431542 at 10 *μ*M (Sigma-Aldrich, St. Louis, MI, USA), and YHJD at 2 mg/mL. Then, the cells were further incubated at 37°C with 5% CO_2_ for 24 h, 48 h, and 72 h, respectively, followed by adding 20 *μ*L of MTT (5 g/L) which was purchased from Sigma-Aldrich (St. Louis, MI, USA) and incubating for 4 h. The supernatant was removed, and then 150 *μ*L of dimethylsulfoxide (DMSO) was added. Absorbance under 490 nm was detected to calculate the inhibition rate. Inhibition rate (% of control) = (1 − absorbance of test sample/absorbance of control) × 100%. The test was repeated three times.

### 2.4. Cell Adhesion Assay

96-well plates were coated with 70 *μ*g/mL FN (BD Biosciences, San Diego, CA) with 30 *μ*L per well, dried on a clean bench, and prepared at 4°C. BGC-803 gastric cancer cells were seeded in culture dishes at 1 × 10^5^/mL. Then, drugs were administered according to their groups: control group, TGF-*β* group, TGF-*β* + YHJD group, SB431542 group, SB431542 + YHJD group, and YHJD group. After 24 h, cells were digested, washed, and resuspended with a drug-containing medium and then seeded in 96-well plates at 1 × 10^5^ per well. The original drug-containing medium was replaced by complete medium every 30 min. Until 120 min, 20 *μ*L of MTT (5 g/L) was added to each well and incubated for 4 h. The supernatant was removed and 150 *μ*L of dimethylsulfoxide was added to each well. Absorbance under 490 nm was detected to calculate the inhibition rate.

### 2.5. Wound-Healing Assay

BGC-803 gastric cancer cells were seeded in 24-well plates at 1 × 10^5^/mL. Once 100% confluence was observed, the cell monolayer was scratched with a pipette tip after washing with phosphate buffer saline (PBS) twice. The relative distance between wound boundaries was recorded by microscopic scanning. Then, drugs were administered according to their groups: control group, TGF-*β* group, TGF-*β* + YHJD group, SB431542 group, SB431542 + YHJD group, and YHJD group. After 24 h culture, the widths of the scratches were photographed for analysis. Wound closure (%) = healing boundary/wound boundary.

### 2.6. Transwell Assay

BGC-803 cells were seeded in culture dishes at 1 × 10^5^/mL and starved in serum-free RPMI-1640 medium for 24 h. Afterwards, they were digested and inoculated at 6 × 10^5^/100 *μ*L in small chambers coated with Matrigel (Millipore, Germany). The chambers were placed in 24-well plates, while 1% FBS was added to the upper chambers and 10% FBS was added to the lower chambers. Drugs were administered according to their groups at the same time: control group, TGF-*β* group, TGF-*β* + YHJD group, SB431542 group, SB431542 + YHJD group, and YHJD group. After 24 h, nonpenetrative cells on the top chamber were wiped away, and the migrating cells were fixed and stained with 0.1% crystal violet. The basement membrane was removed to get five photos of the cells of each group at random selection of microscope fields.

### 2.7. Animal Studies

Gastric cancer MGC-803 cells in the logarithmic growth phase were resuspended and digested at 2.5 × 10^7^/mL. Each mouse was inoculated with 0.2 mL of cell suspension in the armpit. After two weeks, the diameter of the induration grew up to 10 mm. And a successful model was built. The 24 mice were randomly divided into four groups according to the mode of administration: control group (NS 0.2 mL/10 g via gavage), 5-FU group (5-FU 0.1 mL/10 g, 2.5 mg/kg via intraperitoneal injection), high-dose group (YHJD 4 g/mL, 0.2 mL/10 g via gavage), and low-dose group (YHJD 2 g/mL, 0.2 mL/10 g via gavage). They were medicated for 14 days.

### 2.8. Observation on Antitumor Effect

Daily diet, defecation, urination, active status, and tumor growth of the mice were observed. After administration, the short- and long-axis diameters of the tumor were measured every other day. The volume of the tumor was calculated according to Steel formula (V) = (long diameter × short diameter^2^)/2 (cm^3^). Volume inhibition rate (%) = (1 − V_experimental  group_/V_control  group_) × 100%. The mice were sacrificed on the 14th day after administration of drugs.

### 2.9. Immunohistochemistry Assay

Paraformaldehyde-fixed tumor tissue sections (4 mm thick) were mounted on slides, dewaxed, and hydrated. Slides were boiled in 10 mM sodium citrate buffer (pH 6) for 2 min and cooled on bench top for 30 min, followed by incubation in 3% hydrogen peroxide for 15 min and blocking with normal goat serum for 30 min. Sections were incubated with primary antibodies (E-cadherin, N-cadherin, vimentin, Snail, Slug, TGF-*β*R I, p-Smad2, and p-Smad3) and then washed with PBST buffer and incubated with HRP conjugated anti-rabbit or anti-mouse IgG (Boshide Biological Technology Co., Ltd., Wuhan, China). Localization of peroxidase conjugates was revealed using diaminobenzidine tetrahydrochloride solution and chromogen and hematoxylin for counterstaining. Protein expression was observed under an optical microscope. Image-Pro was adopted to analyze each picture to get their integrated optical density (IOD) and area. Mean density (IOD/area) was used to analyze the expression.

### 2.10. Western Blot Analysis

BGC-803 cells were administered with drugs for 24 h according to their groups: control group, TGF-*β* group, TGF-*β* + YHJD group, SB431542 group, SB431542 + YHJD group, and YHJD group. Transplanted tumors in each group (control group, 5-FU group, high-dose group, and Low-dose group) were peeled. The protein lysates from cultured cells and tumor were separated on 10% sodium dodecylsulfate polyacrylamide gel electrophoresis (SDS-PAGE) and transferred to PVDF membranes (Millipore, Billerica, MA, USA). Membranes were blocked with skim milk, followed by incubation with the primary antibodies against E-cadherin, N-cadherin, vimentin, TGF-b, Smad2, Smad3, Snail, Slug, and Twist (Twist was purchased from Abcam, USA, and others were from Cell Signaling Technology, Beverly, MA). Incubation was carried out with horseradish peroxidase-conjugated secondary antibody (Cell Signaling Technology, MA, USA). Signals were observed under the Image Studio version 3.1.4 system.

### 2.11. Statistical Analysis

All data were expressed as means ± standard deviation (SD). One-way ANOVA was used to determine statistically significant differences between groups. Scheffe's test (SPSS 18.0, SPSS Inc., Chicago, IL) was used to correct for multiple comparisons when statistical significance was identified in ANOVA. Statistical significance was set at *P* < 0.05.

## 3. Results

### 3.1. YHJD Time-Dependently Inhibited the Proliferation of Gastric Cancer Cell MGC-803

**Figure 1:**
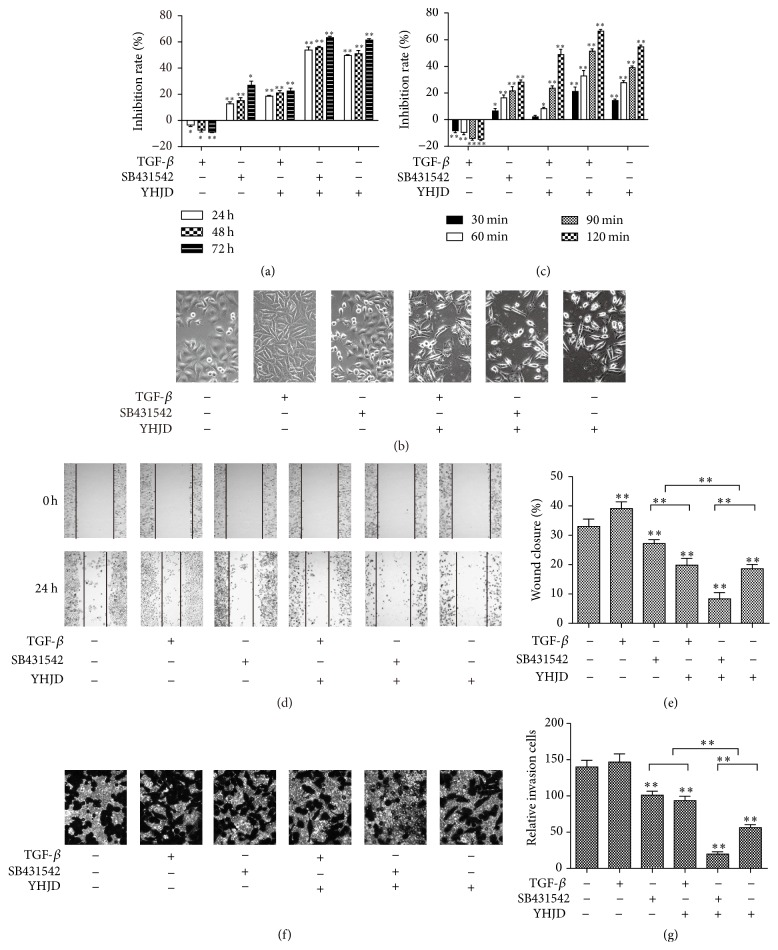
YHJD inhibited gastric cancer MGC-803 cells' proliferation, adhesion, migration, and invasion. (a) TGF-*β* (10 ng/mL), TGF-*β*/Smad (10 *μ*M/L SB431542), and YHJD (2 mg/mL) were administered to cells and cultivated for 24 h, 48 h, and 72 h, and the inhibition rate (%) was determined using MTT assays and was expressed as (1 − absorbance of test sample/absorbance of control) × 100%. (b) YHJD inhibited proliferation of gastric cancer cells and affected cells' morphology. (c) Cell adhesion assay was used to detect MGC-803 cells' adhesion for GC cells. The bar chart was the inhibition rate of cell adhesion of each group at different periods (30, 60, 90, and 120 min). (d) Wound assay detected migration of different groups for MGC-803 cells, which were photographed 24 h after wounding (100x) to record their migration. (e) The bar chart shows wound-healing percentage of gastric cancer MGC-803 cells 24 h after being wounded. (f) Transwell assay detected the effects of each group's invasion effect on MGC-803 cells. Light microscopic images of different concentrations (400x) were taken. (g) The bar chart shows the number of MGC-803 cells that migrated across 8 *μ*m diameter pores to the lower chamber. Data are expressed as the mean ± SD of three experiments (^*∗*^*P* < 0.05 compared with control group, ^*∗∗*^*P* < 0.01 compared with control group).

Cells of the control group was found to be pebble-like under the inverted microscope, and connection between the cells was closer, like that among epithelial cells. Cells of the TGF-*β* group were significantly elongated, like interstitial cells. Compared with the control group, cells of SB431542 group and YHJD group were polygonal, and YHJD time-dependently inhibited the proliferation of MGC-803 cells (*P* < 0.01); its inhibition rate reached 49.65 ± 0.62%, 50.94 ± 2.56%, and 61.49 ± 1.05% at 24 h, 48 h, and 72 h, respectively. The results also showed that, compared with SB431542 group, YHJD more strongly inhibited cells from proliferation (*P* < 0.01), as shown in Figures 1(a) and 1(b).

### 3.2. YHJD Prohibited Adhesion, Migration, and Invasion of MGC-803 Cells

Compared with the control group, TGF-*β* enhanced adhesion and migration of the cells (*P* < 0.01); both SB431542 and YHJD suppressed cells' adhesion, migration, and invasion (*P* < 0.01); compared with SB431542 group, YHJD group and SB431542 + YHJD group had a stronger effect on the inhibition of adhesion and invasion of cells (*P* < 0.01), as shown in Figures 1(c)–1(g).

### 3.3. YHJD Inhibited the Transplanted Tumor Growth in the Mice

**Figure 2:**
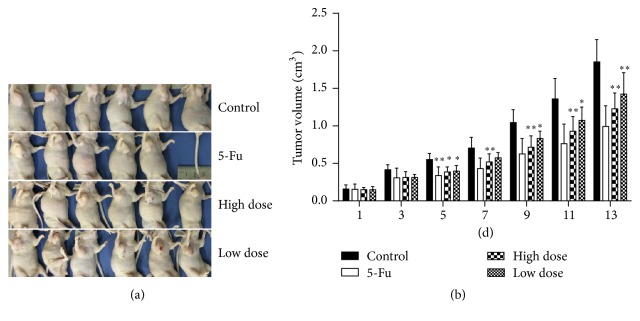
YHJD inhibited the growth of the transplanted tumor. (a) 5 × 10^6^ MGC-803 cells were subcutaneously implanted in the mice. Two weeks after the tumors completely developed, they were divided into control group, 5-FU group, high-dose group, and low-dose group. The tumor was peeled out 14 days after administration. (b). The bar chart shows the change in metastatic tumor volume over medication time. Data are expressed as the mean ± SD of 5 or 6 mice (^*∗*^*P* < 0.05 compared with control group, ^*∗∗*^*P* < 0.01 compared with control group).

The mental state of the animals, intake of food and water, urination, defecation, and other activities were not significantly different between groups. The mice were randomly divided into control group, 5-FU group, high-dose group, and low-dose group. Compared with the control group, the weights of the mice in each group were not significantly different during the experiment (*P* > 0.05). After 5 days of administration, the average tumor volume in the mice of the high-dose group and low-dose group was smaller than that of the control group (*P* < 0.05). At the end of the experiment, compared with the control group, the high-dose group and 5-FU group were found to significantly inhibit the tumor from growing (*P* < 0.01), as shown in Figures 2(a) and 2(b).

### 3.4. YHJD Upregulated the Expression of E-Cadherin through Inhibition of TGF-*β*/Smad Pathway 

**Figure 3:**
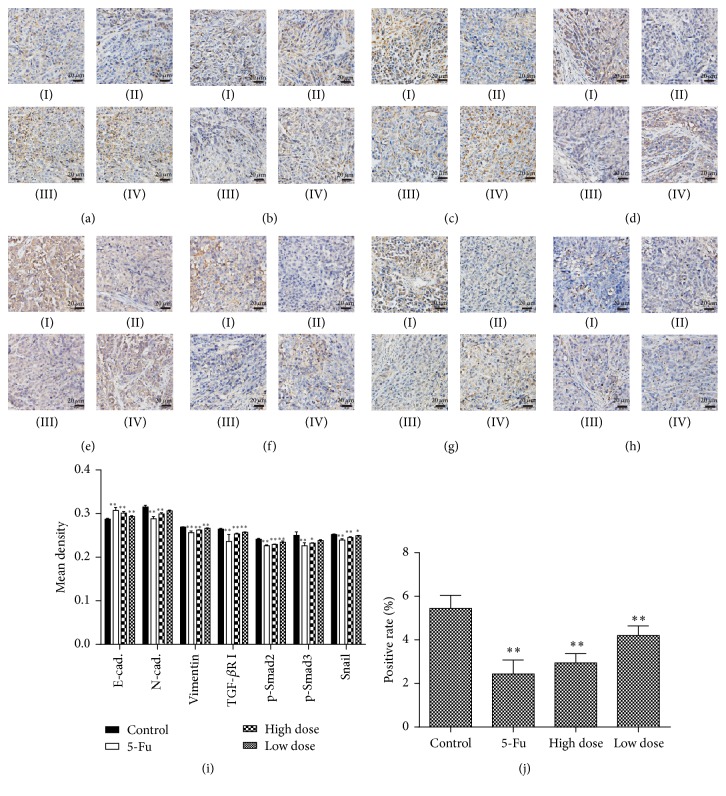
The levels of EMT and TGF-*β*/Smad pathway related proteins in the transplanted tumor were determined by immunohistochemical assay. (a) E-cadherin. (b) N-cadherin. (c) Vimentin. (d) TGF-*β*R I. (e) p-Smad2. (f) p-Smad3. (g) Snail. (h) Slug. Protein of (I) (control group), (II) (5-FU group), (III) (high-dose group), and (IV) (low-dose group) turned brown granular in tumor staining. Positive staining of E-cadherin, N-cadherin, and vimentin focused in the cytoplasm, a few in the envelope, brownish yellow granular. TGF-*β*R I was mainly stained in the cell membrane. p-Smad2 and p-Smad3 in the cytoplasm, a few in the envelope. Snail in the cytoplasm, a few in the nucleus. Slug in the nucleus. (i) The bar chart indicates each molecule's mean density. (j) The bar chart indicates expression positive rate of Slug in metastatic tumor tissue sections. Data are expressed as the mean ± SD of three experiments (^*∗*^*P* < 0.05 compared with control group, ^*∗∗*^*P* < 0.01 compared with control group).

Immunohistochemistry showed that, compared with the control group, E-cadherin level increased (*P* < 0.05) in the high-dose group, low-dose group, and 5-FU group, while levels of N-cadherin, Twist, vimentin, TGF-*β*R I, p-Smad2, p-Smad3, Snail, and Slug declined (*P* < 0.05), as shown in Figures 3(a)–3(j).

### 3.5. TGF-*β* Inhibited the Expression of E-Cadherin through Activation of TGF-*β*/Smad Pathway, Which Can Be Abolished by YHJD

**Figure 4:**
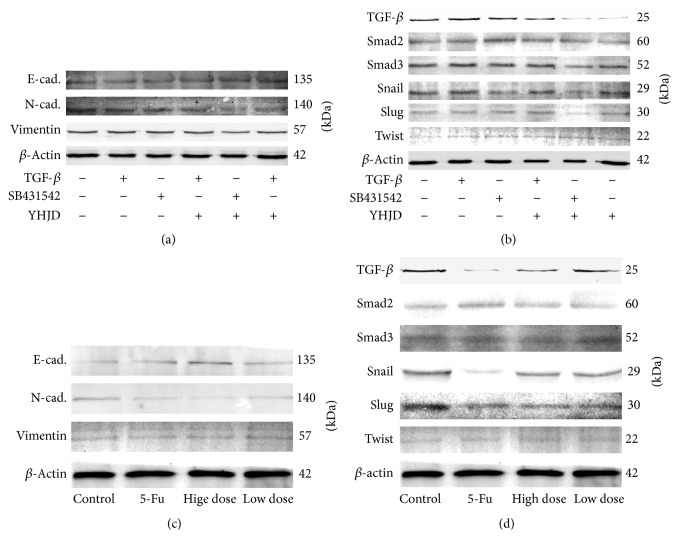
After MGC-803 was administered for 24 h, the levels of (a) EMT (E-cadherin, N-cadherin, and vimentin) and (b) TGF-*β*/Smad pathway (TGF-*β*, Smad2, Smad3, Twist, Snail, and Slug) related proteins were determined by western blot. (c-d) The protein expression in the transplanted tumor about EMT (E-cadherin, N-cadherin, and vimentin) and TGF-*β*/Smad pathway (TGF-*β*, Smad2, Smad3, Snail, Slug, and Twist). *β*-Actin was taken as a loading control.

Western blot showed that, in the cell assay, compared with the control group, E-cadherin of TGF-*β* group declined, while the level of N-cadherin, vimentin, TGF-*β*, and Snail increased. Compared with the control group, N-cadherin, vimentin, and Snail of SB431542 group declined. Compared with the control group and TGF-*β* group, E-cadherin of YHJD group and SB431542 + YHJD group increased, while the level of N-cadherin, vimentin, TGF-*β*, and Snail declined. No significant difference in each factor's expression was found between YHJD group and SB431542 + YHJD group. No significant difference in Smad2, Smad3, Slug, and Twist expression was found between groups, as shown in Figures 4(a) and 4(b). In the transplanted tumor, compared with the control group, E-cadherin was upregulated in the high-dose group, low-dose group, and 5-FU group, while the level of N-cadherin, vimentin, TGF-*β*, Snail, and Slug went downwards. No significant difference in Smad2, Smad3, Twist, and vimentin was found between groups, as shown in Figures 4(c) and 4(d).

## 4. Discussion

The recurrence and distant metastasis of gastric cancer, as one of the world's top 10 malignant tumors, have become a great threat to human health and a leading cause of mortality, and thus effective strategies are required. Traditional Chinese medicine has absolutely a big advantage in the treatment of various intractable diseases. At present, it is attracting more and more attention because it is multitargeted and treats both the tip and root of an illness, reduces toxins at the same time, and enhances efficacy, especially in the treatment of cancer. The Oncology Department of Jiangsu Provincial Hospital of TCM adopted an important treatment principle of replenishing qi, resolving stasis, and removing toxins for recurrence and metastasis of gastric cancer in long-term clinical practice. As an adjuvant therapy for gastric cancer patients after surgery, YHJD can improve the condition of qi deficiency and toxic stasis, enhance the effect of and patient's sensitivity to chemotherapy, and thus reduce the risk of recurrence and metastasis and improve the quality of life of patients. So far, it has gained a great therapeutic value. However, the mechanism of the effects of YHJD on the metastasis of gastric cancer has not been understood.

Recent studies have revealed that gastric carcinoma metastasis is closely related to epithelial-mesenchymal transition (EMT) [10, 11]. Transforming growth factor-*β* (TGF-*β*) may play a critical role in EMT of malignant tumors belonging to epithelial origin [7, 12, 13]. Overexpression of TGF-*β* activates TGF-*β*/Smad pathway, and TGF-*β* binds to TGF-*β* receptors I and II to activate phosphorylated Smad2/3, thereby activating Smad4 to transport TGF-*β*/Smad signal into the cell nucleus to adjust EMT [14]. Therefore, in order to clarify the mechanism of TCM's inhibition of gastric cancer, the present study, based on TGF-*β*/Smad pathway, observed YHJD's efficacy on gastric cancer. SB431542 has been shown to selectively block TGF-*β*/Smad pathway through blocking the phosphorylation of Smad2 [15, 16]. Since we use the gain and loss of function experiment to further verify the effect of YHJD, SB431542 has been shown to suppress the TGF-*β*-induced EMT and proliferation of gastric cancer cells in humans. Therefore, we use TGF-*β* as the activator of TGF-*β*/Smad pathway while SB431542 is used as the inhibitor. Cell experiments showed that, related to the control group, proliferation, adhesion, and migration of gastric cancer cells in TGF-*β* group were improved, while proliferation, adhesion, and migration of gastric cancer cells in SB431542 group were reduced. The results indicated that activation of TGF-*β*/Smad pathway promoted the proliferation and metastasis of gastric cancer cells, consistent with literature reports. Compared with the control group,* YHJD time-dependently inhibited the proliferation of gastric cancer cell MGC-803. YHJD can also prohibit adhesion, migration, and invasion of MGC-803 cells, *which is in accordance with its clinical efficacy. Compared with TGF-*β* group, TGF-*β* + YHJD group can revert TGF-*β*'s promotion effect on gastric cancer cells' proliferation, adhesion, and migration, indicating that this pathway might play an important role in YHJD's inhibition of gastric cancer development. Compared with SB431542 group, YHJD group and SB431542 + YHJD group had a stronger ability to inhibit cell proliferation and metastasis, which may be related to YHJD's apoptosis induced function. That is, YHJD probably interfered with other mechanisms to inhibit gastric cancer as well, which was worthy of further studies. Our animal experiments illustrated that, compared with the control group, YHJD inhibited the tumor's growth in the mice, which was in accordance with clinical and experimental results.

Loss of epithelial polarity and gain of interstitial cells are characteristic marks of EMT [17, 18], including loss of expression and function of E-cadherin protein and other epithelial markers and overexpression of interstitial cell markers like N-cadherin and vimentin. Meanwhile, transcription factors like Snail, Slug, and Twist which negatively regulate E-cadherin were upregulated [19]. Therefore, local infiltration and distant metastasis of the tumor were promoted. TGF-*β*/Smad pathway is the key to TGF-*β*-induced EMT. Inhibition of TGF-*β*/Smad pathway can reduce tumor invasion and metastasis [20]. In our cell experiments, western blot assay found that a high expression of TGF-*β* was found in TGF-*β* group, while TGF-*β* + YHJD group can decrease its expression, indicating that this pathway might play an important role in YHJD's inhibition of gastric cancer development, which is in accordance with cell behavior experiments. YHJD can reduce the expression of TGF-*β*, prevent TGF-*β*/Smad pathway from being activated, and thereby inhibit EMT by upregulating EMT-related phenotypes of E-cadherin and downregulating N-cadherin, vimentin, Snail, and Slug. In animal experiments, immunohistochemistry and western blot assay found that YHJD reduced the expression of TGF-*β*, p-Smad2, and p-Smad3 and downregulated N-cadherin and vimentin. This was in line with the results of cell experiments. Accordingly, we believe that YHJD may inhibit gastric cancer metastasis by inhibiting TGF-*β*/Smad's activation. But there is still a possibility that YHJD inhibits EMT through other pathways or regulates other factors to activate TGF-*β*/Smad pathway. This undoubtedly suggests that YHJD has multitarget antitumor activity, which is of great significance for the treatment of cancer.

Although there were important results in this study, there were also some limitations. For example, TGF-*β*/Smad pathway is the only pathway we use to clarify YHJD's transfer resistance mechanism. More experiments, such as apoptosis related assays, are required to further identify the true active components and biological functions of this decoction.

## 5. Conclusions

Based on TGF-*β*/Smad's signaling pathway, the present study explored the mechanism of YHJD in gastric cancer metastasis. It was found that YHJD could inhibit EMT through TGF-*β*/Smad pathway. Moreover, YHJD has multitarget antitumor activity, which is of scientific significance to the clinical practice and development of new drugs.
