# Corrigendum to “Yiqi Huayu Jiedu Decoction Inhibits the Invasion and Metastasis of Gastric Cancer Cells through TGF-*β*/Smad Pathway”

**DOI:** 10.1155/2021/4589203

**Published:** 2021-01-08

**Authors:** Ting-Ting Wu, Jun Lu, Pei-Qiu Zheng, Shen-Lin Liu, Jian Wu, Wei Sun, Qing-Min Sun, Nai-Xia Ma, Xue-Lian Ding, Min Chen, Xi Zou

**Affiliations:** ^1^The Affiliated Hospital of Nanjing University of TCM, Jiangsu Province Hospital of TCM, Nanjing 210029, China; ^2^No. 1 Clinical Medical College, Nanjing University of Chinese Medicine, Nanjing, Jiangsu 210023, China

In the article titled “Yiqi Huayu Jiedu Decoction Inhibits the Invasion and Metastasis of Gastric Cancer Cells through TGF-*β*/Smad Pathway” [1], the clinical rationale for the study was not adequately supported because the statement that “Clinical study on large samples has shown that YHJD can obviously improve the quality of life of patients and prolong their survival” was unreferenced. The study was based on the outcomes of the project “Study on the individualized treatment based on Jianpi Yangwei for postoperative gastric cancer recurrence and metastasis (健脾养胃法为主的个体化治疗对胃癌术后复发转移干预方案的研究)” (200807022), which was funded by the National Administration of Traditional Chinese Medicine and ended in 2016. One of the authors, Dr. Liu, published a prospective cohort study on YHJD in gastric cancer in 2014 [2], updated in English in 2019 [3].
